# Computed tomography of the occipital bone for sex estimation in Turks

**DOI:** 10.1590/1806-9282.20260627

**Published:** 2026-10-12

**Authors:** Esra Arslan, Veysel Osman Soğukpınar, Melahat Kul, Yaşar Bilge

**Affiliations:** 1 Ministry of Justice, Council of Forensic Medicine, The Group Presidency of Forensic Medicine – Ankara, Türkiye.; 2 Ministry of Justice, Council of Forensic Medicine, The Group Presidency of Forensic Medicine – Van, Türkiye.; 3 Ankara University, Faculty of Medicine, Department of Internal Medicine, Division of Radiology – Ankara, Türkiye.; 4 Ankara University, Faculty of Medicine, Department of Internal Medicine, Division of Forensic Medicine – Ankara, Türkiye.

**Keywords:** Sex determination by skeleton, Forensic anthropology, Tomography, Skull, Anthropometry

## Abstract

**OBJECTIVE:**

Sex estimation is a fundamental component of forensic identification when DNA analysis is not feasible. Because cranial morphology and sexual dimorphism vary among populations, population-specific standards are required. However, occipital bone morphometric data for Turkish adults remain limited. Therefore, the aim of this study was to evaluate the reliability of occipital bone measurements obtained from cranial computed tomography for sex estimation in Turkish adults.

**METHODS:**

Three-dimensional cranial computed tomography images of 384 individuals (192 females, 192 males), aged 18–65, obtained between 01/01/2010 and 31/12/2021, were retrospectively analyzed. Nine anatomical distances on the occipital bone were measured, and their discriminative roles in sex estimation were assessed using logistic regression analysis. Additionally, inter-observer agreement was evaluated by comparing measurements from two independent observers.

**RESULTS:**

The combined analysis of all measurements yielded a sex prediction accuracy of 82.8% for females and 80.2% for males. Foramen magnum width, occipital condyle length, occipital condyle width, and external occipital protuberance depth were identified as the most discriminative variables. A model combining these four variables achieved classification accuracies of 83.3% for females and 79.2% for males, with an overall accuracy of 81.3%. The full model including all nine measurements showed an overall accuracy of 81.5%. High intra- and inter-observer agreement was observed.

**CONCLUSION:**

Computed tomography-based morphometric analysis of the occipital bone is a reliable method for sex estimation in forensic medicine. These results support the development of population-specific reference data for the Turkish population.

## INTRODUCTION

Identity encompasses the biological and sociological characteristics that distinguish an individual; determining these characteristics constitutes identification. In forensic and social contexts, identification applies to both living and deceased persons and is categorized as forensic identity and medical identity. Forensic identity includes demographic data (sex, date and place of birth, parents’ names) and official documentation such as identity cards, driver's licenses, and passports. Medical identity refers to physical and anatomical features including stature, body weight, age, pigmentation traits, facial morphology, scars, surgical marks, tattoos, birthmarks, skeletal anomalies, and dental characteristics^1^.

In forensic medicine, sex estimation is a fundamental component of constructing the biological profile of unidentified individuals^2^. Accurate sex determination narrows missing person searches and guides assessment of age, ancestry, and stature. Although DNA profiling provides high accuracy, it may be limited by cost, time requirements, or lack of comparative samples. In such cases, anthropological and morphometric methods serve as important alternatives, particularly when rapid results are required^3^.

Sexual dimorphism of the human skeleton is well established, and the cranium is one of the most extensively studied regions for sex estimation. Shim et al.^4^ reported approximately 93% accuracy using three-dimensional cranial metric analysis. Recent research trends indicate that metric methods are increasingly prioritized over non-metric (morphological) approaches in forensic sex estimation due to their quantifiable and replicable nature, which tends to reduce observer-dependent variability^5^. In fragmented remains, the pelvis and cranium are generally regarded as the most informative skeletal elements for sex estimation, although other bones have also been investigated^3,6,7^. Advances in computed tomography (CT) and magnetic resonance imaging (MRI) enable rapid, objective, and three-dimensional evaluation of skeletal structures. Moreover, cranial CT allows detailed morphometric assessment without the need for direct skeletal examination and has become increasingly available in both clinical and forensic settings. Since cranial morphology and the degree of sexual dimorphism differ among populations because of genetic, environmental, and geographical factors, population-specific reference standards are essential for accurate sex estimation^8^. However, studies evaluating occipital bone morphometry in the Turkish population remain limited. Therefore, the present study aimed to investigate sexual dimorphism in linear measurements of the occipital bone using cranial CT and to evaluate their reliability for sex estimation in Turkish adults.

## METHODS

### Study design

This descriptive cross-sectional study assessed the applicability of occipital bone linear measurements on CT images for sex estimation in individuals aged 18–65 years.

Sample size was determined based on planned statistical analyses and literature-reported effect sizes (0.15, 0.30, 0.40, and 0.50) with 95% power, requiring a minimum of 384 participants. Accordingly, cranial CT scans of 384 individuals (192 females and 192 males) aged 18–65 years, obtained at the Department of Radiology, Ankara University Faculty of Medicine between 01/01/2010 and 31/12/2021, were included. Participants were evenly distributed across age subgroups (18–24, 25–30, 31–39, 40–49, 50–59, and 60–65).

Exclusion criteria comprised prior cranial base surgery, trauma-related deformity, and tumors, masses, or hydrocephalus involving the cranial base.

Demographic and clinical data were retrieved from the AVICENNA hospital information management system. All CT images were evaluated collaboratively by radiology and forensic medicine specialists.

### Radiological evaluation

All CT images were analyzed using VITREA advanced visualization software (v7.5.0.510, Toshiba) with multiplanar and three-dimensional reconstructions. Examinations were performed using Somatom Emotion (Siemens, 16-slice) and Prime Aquillon (Toshiba, 80-slice) scanners. CT acquisition parameters included a slice thickness of 0.5–0.625 mm, tube current of 140–300 mA, tube voltage of 120–140 kVp, and pitch values of 0.348–0.637.

Linear measurements of the occipital bone were obtained in millimeters (mm) and recorded in centimeters (cm). These measurements included: external occipital protuberance depth (EOPD) (perpendicular distance from the most prominent point to the bone base), foramen magnum width (FMW) (maximum width measured perpendicular to the midline), foramen magnum length (FML) (maximum distance between basion and opisthion), inion-opisthion length (IOL) (external measurement), lambda-inion length (LIL) (external measurement), lambda-opisthion length (LOL) (external measurement), occipital condyle width (OCW) (maximum width perpendicular to the long axis of the condyle), occipital condyle length (OCL) (maximum length along the long axis of the condyle), and interasterion distance (IAD) (external distance between the right and left asterion). Representative three-dimensional and axial cranial CT images illustrating the anatomical landmarks and morphometric measurements used in the present study are shown in Figure 1.

**Figure 1:**
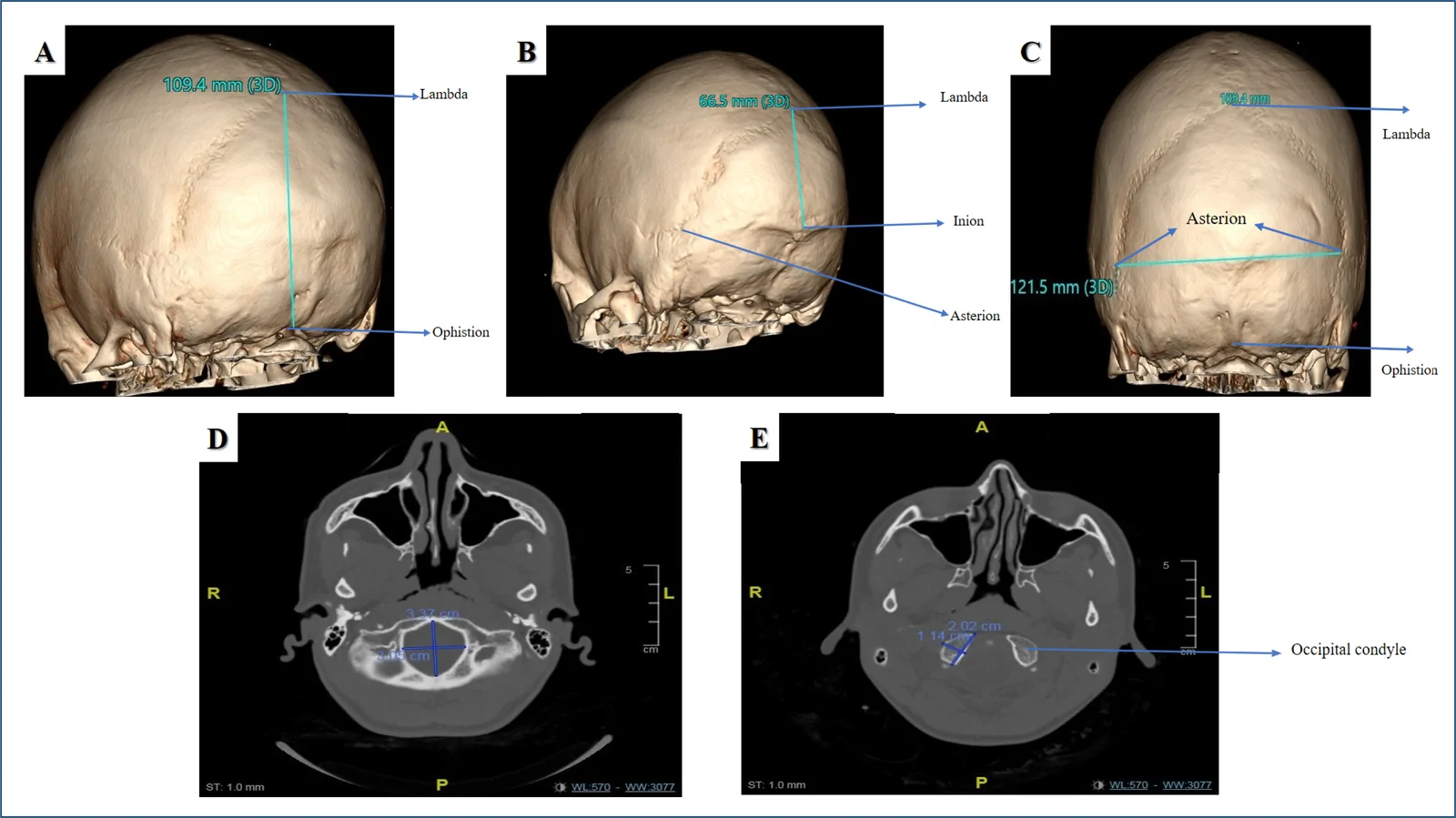
Representative three-dimensional and axial cranial computed tomography images illustrating the anatomical landmarks and morphometric measurements used in this study. **(A)** Lambda–opisthion length. **(B)** Lambda–inion length. **(C)** Interasterionic distance. **(D)** Foramen magnum length and foramen magnum width.**(E)** Occipital condyle length and occipital condyle width. A: anterior; P: posterior; R: right; L: left.

### Statistical analysis

Statistical analyses were performed using statistical package for the social sciences (SPSS) version 26.0 (Armonk, New York, USA); p<0.05 was considered statistically significant.

Continuous variables were expressed as mean±standard deviation and categorical variables as number and percentage. Distribution was assessed using histograms, probability plots, and the D’Agostino-Pearson, Kolmogorov-Smirnov, and Shapiro-Wilk tests. Outliers in non-normally distributed data were removed, and Winsorization was applied when required. Homogeneity of variances was evaluated using the Levene test.

Between-sex comparisons were performed using the independent t-test or Welch t-test; the Kruskal-Wallis test was used for non-normal distributions, and one-way analysis of variance (ANOVA) for normally distributed comparisons involving more than two groups.

Intra- and inter-observer agreement were assessed using the Paired t-test and Wilcoxon Paired test. The chi-square test and Binary Logistic Regression were used to determine diagnostic performance in sex discrimination, and model fit was evaluated with the Omnibus test.

### Ethics committee approval

Approval for this study was obtained from the Ankara University Faculty of Medicine Human Research Ethics Committee (decision number İ04-275-23, dated 10/05/2023), and the principles of the Declaration of Helsinki were taken into consideration.

## RESULTS

A total of 384 individuals (192 males, 192 females; 18–65 years) were included.

Except for IOL (p=0.05), all occipital measurements (LOL, LIL, OCL, OCW, EOPD, IAD, FML, and FMW) showed significant sex differences (p<0.001). Welch's t-test was applied to LOL, EOPD, and IAD due to variance heterogeneity; the independent samples t-test was used for the remaining variables. For all significant parameters, females exhibited lower mean values than males (Table 1).

**Table 1:** Cranial (occipital bone) measurement values by sex and statistical comparison results.

Measurements (parameters)	Levene test of variance (p)	Levene variance homogeneity (+/-)	Test type	t-value	p-value	Average difference (cm)	Average value for women (cm)	Average value for men (cm)
IOL	0.88	+	Independent t-test	1.95	0.05	0.1243	4.74	4.87
LOL	<0.05	-	Welch t-test	5.82	<0.001	0.4174	9.91	10.33
LIL	0.50	+	Independent t-test	5.69	<0.001	0.4900	6.41	6.90
OCL	0.82	+	Independent t-test	10.50	<0.001	0.2084	1.72	1.92
OCW	0.12	+	Independent t-test	7.79	<0.001	0.1027	0.87	0.97
EOPD	0.02	-	Welch t-test	10.77	<0.001	0.3435	1.07	1.42
IAD	0.02	-	Welch t-test	9.27	<0.001	0.5016	11.09	11.59
FML	0.43	+	Independent t-test	9.62	<0.001	0.2670	3.33	3.59
FMW	0.23	+	Independent t-test	8.46	<0.001	0.2081	2.88	3.08

OCL: occipital condyle length; OCW, occipital condyle width; EOPD, external occipital protuberance depth; LOL, lambda-opisthion length; LIL, lambda-inion length; IOL, inion-opisthion length; FML, foramen magnum length; FMW, foramen magnum width; IAD, interasterion distance.

Binary Logistic Regression including all variables (IOL, LOL, LIL, OCL, OCW, EOPD, IAD, FML, and FMW) demonstrated overall model significance (Omnibus p<0.001). The model correctly classified 82.8% (n=159) of females and 80.2% (n=154) of males, with an overall accuracy of 81.5% (n=313). OCL, OCW, EOPD, and FMW were significant predictors; increasing values were associated with male sex (Table 2).

**Table 2:** Sex estimation based on cranial (occipital bone) measurements and binary logistic regression results.

Independent variable parameters (measurements)	Regression coefficient	Standard error	Wald statistic (chi-square)	Degree of freedom	Significance level (p)	Probability ratio
LOL	0.079	0.133	0.352	1	0.553	1.082
LIL	0.051	0.039	1.686	1	0.194	1.052
IOL	-0.037	0.063	0.351	1	0.553	0.964
OCL	3.332	0.823	16.377	1	<0.001	28.005
OCW	3.510	1.230	8.143	1	0.004	33.441
EOPD	3.567	0.527	45.740	1	<0.001	35.418
IAD	-0.026	0.071	0.138	1	0.711	0.974
FML	-0.016	0.084	0.037	1	0.848	0.984
FMW	4.199	0.660	40.422	1	<0.001	66.614
Fixed	-26.740	3.127	73.119	1	<0.001	0.000

OCL: occipital condyle length; OCW, occipital condyle width; EOPD, external occipital protuberance depth; LOL, lambda-opisthion length; LIL, lambda-onion length; IOL, inion-opisthion length; FML, foramen magnum length; FMW, foramen magnum width; IAD, interasterion distance.

Seven additional measurement combinations were tested. All models were statistically significant. The highest accuracy (81.3%) was obtained with the OCL-OCW-FMW-EOPD model, whereas the lowest (61.2%) was observed for LOL-LIL-IOL. In all models, higher values of significant variables were positively associated with male sex (Table 3).

**Table 3:** Logistic regression analysis of cranial (occipital bone) metric parameters (measurements) in sex differentiation.

Combination models	Omnibus test (p)	Correct classification "%" (female/male/overall average)	Correct classification "n" (female/male/total)	Significant parameters (measurements)	Comment
OCL, OCW, EOPD	<0.001	79.7%/76%/77.9%	153/146/299	OCL, OCW, EOPD	An increase in all measurements indicates a transition toward the male sex.
LOL, LIL, IOL	0.004	64.1%/58.3%/61.2%	123/112/235	Only LOL	An increase in the LOL measurement indicates a transition toward the male sex.
FML, FMW	0.004	67.2%/67.7%/67.4%	129/130/259	Only FMW	An increase in the FMW measurement indicates a transition toward the male sex.
OCL, OCW, IAD	<0.001	74%/71.4%/72.7%	142/137/279	OCL, OCW	An increase in the OCL and OCW measurements indicates a transition toward the male sex.
LOL, IAD	<0.001	72.4%/66.7%/69.5%	139/128/267	LOL, IAD	An increase in both measurements indicates a transition toward the male sex.
OCL, OCW, FML, FMW	<0.001	75.5%/71.4%/73.4%	145/137/282	OCL, OCW, FMW	The FML measurement is not significant; increases in the others indicate a transition toward the male sex.
OCL, OCW, FMW, EOPD	<0.001	83.3%/79.2%/81.3%	160/152/312	OCL, OCW, FMW, EOPD	An increase in all measurements indicates a transition toward the male sex.

OCL: occipital condyle length; OCW: occipital condyle width; EOPD, external occipital protuberance depth; LOL, lambda-opisthion length; LIL, lambda-inion length; IOL, inion-opisthion length; FML, foramen magnum length; FMW, foramen magnum width; IAD, interasterion distance.

Inter- and intra-observer reliability was assessed in 48 cases evaluated independently by a forensic medicine faculty member and a radiology specialist. After normality testing, the Paired t-test (parametric variables) and Wilcoxon Paired test (IOL) were applied. No significant inter-observer differences were detected for any measurement (p>0.05), indicating high measurement consistency.

## DISCUSSION

In forensic practice, identification is the primary step for both living and deceased individuals, and sex estimation constitutes its foundation. Accurate sex determination directly affects the reliability of the remaining components of the biological profile. Although DNA analysis remains the gold standard through X and Y chromosome differentiation^9^, morphological and metric methods are indispensable when DNA is unavailable, particularly in degraded or limited samples^10^. Therefore, standardized anthropological methods applicable to skeletal remains should be maintained as complementary tools in forensic identification.

Bone tissue may be the only available source of genetic material in certain cases due to its relative capacity to preserve DNA^11^. However, environmental and physical factors may compromise DNA integrity. Acidic soil conditions, chemical exposure, and high temperatures—such as in fire-related events—can cause denaturation and severe degradation^12^. Consequently, DNA extraction from bone is technically complex and does not consistently yield usable results. These limitations reinforce the necessity of morphometric approaches when genetic analysis is restricted.

In this study, the diagnostic value of the occipital bone was evaluated in forensic scenarios involving limited material, degraded remains, or time constraints. In mass fatalities, commingled remains, or fragmented skeletons, the pelvis, long bones, and cranium are primary structures for sex estimation^13^. Using nine linear measurements from anatomical landmarks of the occipital bone—one of the most durable cranial regions—an overall accuracy of 81.5% was achieved. Previous studies based solely on foramen magnum or occipital measurements reported accuracies ranging from 66.5 to 88%^7,14–17^, while models incorporating additional cranial parameters reached 87%^18^. These findings collectively support the diagnostic relevance of occipital bone metrics in sex estimation and their integration into routine forensic protocols.

Kirisattayakul et al.^19^ demonstrated sexual dimorphism of the external occipital protuberance (EOP) in a Thai population using cranial CT. In the present study, EOP was assessed metrically through EOPD (1.07 cm in females; 1.42 cm in males), confirming dimorphism while emphasizing the objectivity of quantitative evaluation. The growing application of three-dimensional imaging in forensic anthropology enhances measurement precision, reproducibility, and efficiency, enabling retrospective analyses and rapid identification.

Except for IOL, all occipital parameters showed significant sexual dimorphism. Similar findings were reported by Ekizoglu et al.^18^ in a Turkish cohort (87% accuracy), Kelley and Tallman^20^ (81–87% accuracy), and Meral et al.^21^ (up to 88% accuracy). Compared with the 81.5% overall and 81.3% best-model accuracies obtained here, occipital measurements demonstrate performance comparable to established cranial metric methods. High intra- and inter-observer agreement further confirms methodological reliability.

Foramen magnum-based sex estimation shows population variability. Gapert et al.^15^ and Suazo et al.^16^ identified FMW as the most discriminative parameter, whereas Raghavendra Babu et al.^7^ reported higher dimorphism for FML. In the present study, FMW remained statistically significant, while FML showed lower predictive value (overall 67.4% when analyzed together). These inconsistencies likely reflect geographic, genetic, and methodological differences. Thus, while foramen magnum metrics may be useful in specific populations, standardized multicenter studies are necessary to enhance generalizability.

Population variability is also evident in other cranial regions. Zheng et al.^22^ reported sex estimation accuracies of 62.4% (Bosnian), 75.2% (Chinese), and 78.4% (Nepalese) using frontal sinus morphometry, while a Chinese study reported 76.6% accuracy^23^. These findings emphasize the influence of genetic and environmental factors on cranial dimorphism and highlight the need for population-specific reference data. The present study contributes such data for Turkish adults.

Occipital condyle morphometry has demonstrated moderate-to-high diagnostic performance. Gapert et al.^14^ reported accuracies of 69.2–76.7% using condylar measurements, while a systematic review by Tsiouris et al.^24^ confirmed the value of CT-based three-dimensional evaluation of this region. In the present study, OCL and OCW were significant predictors and achieved 81.3% accuracy when combined with FMW and EOPD. Similarly, Hoover and Thomas^17^ reported 71% accuracy using combined occipital parameters, including EOPD and FMW, reinforcing their consistent discriminative value.

The relative resistance of occipital structures to trauma, burning, and decomposition further enhances forensic applicability. Additionally, the absence of significant measurement differences between observers in this study confirms high reproducibility. MRI-based studies have likewise demonstrated strong intra- and inter-observer agreement for cranial base parameters^25^, supporting the reliability of advanced imaging–based morphometry.

From a practical perspective, the proposed CT-based morphometric approach may serve as a valuable adjunct in forensic identification, particularly when highly sexually dimorphic skeletal elements, such as the pelvis, are unavailable. Because cranial CT examinations are routinely performed in many clinical settings, retrospective imaging datasets may provide an accessible source for developing and validating population-specific forensic standards. Although the proposed model should not replace comprehensive anthropological or genetic analyses, it may contribute to the biological profile as a complementary method when conventional approaches are limited.

The present study has several strengths. First, it included a relatively large and sex-balanced sample of Turkish adults. Second, all measurements were obtained from three-dimensional CT images using standardized anatomical landmarks, thereby reducing measurement variability. Third, intra- and inter-observer reliability analyses demonstrated high reproducibility of the measurements. Finally, the evaluation of multiple occipital parameters within the same predictive model provides comprehensive reference data for forensic applications in the Turkish population.

This study also has several limitations. First, its retrospective single-center design may limit the generalizability of the findings. Second, the study population consisted only of Turkish adults and may not fully represent all regional and ethnic variations within the country. Third, no external validation cohort was available to evaluate the predictive model independently. Therefore, future multicenter studies including larger and more diverse populations are needed to externally validate the proposed model and improve its generalizability.

Overall, the findings of the present study indicate that CT-based occipital bone morphometry is a reproducible, objective, and non-invasive approach that may assist sex estimation in Turkish adults. Although predictive performance remains population-dependent, the proposed model provides valuable complementary information for forensic identification. Future multicenter studies with external validation cohorts are warranted to further improve the robustness and generalizability of these population-specific reference data.

## CONCLUSION

CT-based occipital morphometry represents a reliable, objective, and non-invasive complementary tool for sex estimation in Turkish adults. FMW, OCL, OCW, and EOPD demonstrated significant sexual dimorphism, with the four-parameter logistic regression model achieving an accuracy of 81.3%, suggesting that a reduced model may be sufficient for practical forensic applications. The postmortem durability of the occipital region and the high inter-observer agreement further support its forensic utility. These findings provide population-specific reference data for Turkish adults; however, multicenter studies with independent validation cohorts are warranted to enhance the generalizability of the proposed model.

## Data Availability

The datasets generated and/or analyzed during the current study are available from the corresponding author upon reasonable request.

## References

[1] Dokgöz H (2019). Forensic medicine and forensic sciences [Turkish].

[2] Dokgöz H (2019). Forensic medicine and forensic sciences [Turkish].

[3] Zhang M (2024). The application of forensic imaging to sex estimation: focus on skull and pelvic structures. Perspect Legal Forensic Sc.

[4] Shim YT, Jeong YH, Aum N, Ha HI, Choi M, Hyun JY (2024). A new metric method for sex estimation using three-dimensional imaging of the nuchal crest. Anat Cell Biol.

[5] Ferrell MJ, Schultz JJ, Adams DM (2024). Sex estimation research trends in forensic anthropology between 2000 and 2022 in five prominent journals. J Forensic Sci.

[6] Pilli E, Palamenghi A, Cattaneo C (2025). Forensic skeletal and molecular anthropology face to face: combining expertise for identification of human remains. Ann N Y Acad Sci.

[7] Raghavendra Babu YP, Kanchan T, Attiku Y, Dixit PN, Kotian MS (2012). Sex estimation from foramen magnum dimensions in an Indian population. J Forensic Leg Med.

[8] Dereli AK, Zeybek V, Sagtas E, Senol H, Ozgul HA, Acar K (2018). Sex determination with morphological characteristics of the skull by using 3D modeling techniques in computerized tomography. Forensic Sci Med Pathol.

[9] Latham KE, Miller JJ (2018). DNA recovery and analysis from skeletal material in modern forensic contexts. Forensic Sci Res.

[10] Gupta A, Billings BK, Hummel S, Grosskopf B (2022). Evaluating morphological methods for sex estimation on isolated human skeletal materials: comparisons of accuracies between German and South African skeletal collections. Forensic Sci.

[11] Jakubowska J, Maciejewska A, Pawłowski R (2012). Comparison of three methods of DNA extraction from human bones with different degrees of degradation. Int J Legal Med.

[12] Bhoyar L, Mehar P, Chavali K (2024). An overview of DNA degradation and its implications in forensic caseworks. Egypt J Forensic Sci.

[13] Yasar Teke H, Ünlütürk Ö, Günaydin E, Duran S, Özsoy S (2018). Determining gender by taking measurements from magnetic resonance images of the patella. J Forensic Leg Med.

[14] Gapert R, Black S, Last J (2009). Sex determination from the occipital condyle: discriminant function analysis in an eighteenth and nineteenth century British sample. Am J Phys Anthropol.

[15] Gapert R, Black S, Last J (2009). Sex determination from the foramen magnum: discriminant function analysis in an eighteenth and nineteenth century British sample. Int J Legal Med.

[16] Suazo GIC, Russo PP, Zavando MDA, Smith RL (2009). Sexual dimorphism in the foramen magnum dimensions. Int J Morphol.

[17] Hoover KC, Thomas GP (2022). Sexual dimorphism and biomechanical loading in occipital bone morphological variation. Am J Hum Biol.

[18] Ekizoglu O, Hocaoglu E, Inci E, Can IO, Solmaz D, Aksoy S (2016). Assessment of sex in a modern Turkish population using cranial anthropometric parameters. Leg Med (Tokyo).

[19] Kirisattayakul W, Boonruengudom G, Chalardpongpun P, Duangchit S, Poodendaen C, Aorachon P (2025). Forensic morphometry and prevalence of external occipital protuberance types in Northeastern Thais: CT and dry skull evidence for sex and age estimation. Forensic Sci.

[20] Kelley SR, Tallman SD (2022). Population-inclusive assigned-sex-at-birth estimation from skull computed tomography scans. Forensic Sci.

[21] Meral O, Meydan R, Toklu BB, Kaya A, Karadayi B, Acar T (2022). Estimation of sex from computed tomography images of skull measurements in an adult Turkish population. Acta Radiol.

[22] Zheng B, Zhong Y, Al-Worafi NA, Liu Y (2023). The dimensional and morphological assessment of the frontal sinus in sex estimation among different populations. Head Face Med.

[23] Luo H, Wang J, Zhang S, Mi C (2018). The application of frontal sinus index and frontal sinus area in sex estimation based on lateral cephalograms among Han nationality adults in Xinjiang. J Forensic Leg Med.

[24] Tsiouris C, Triantafyllou G, Karangeli N, Botis GG, Papadopoulos-Manolarakis P, Kalamatianos T (2025). Morphometric assessment of occipital condyles and foramen magnum reveals enhanced sexual dimorphism detection via 3D imaging: a systematic review and meta-analysis utilizing classification and regression trees. Diagnostics (Basel).

[25] Kizilgoz V, Aydin S, Aydemir H, Keles P, Kantarci M (2024). Interobserver and intraobserver reliability of skull base angles measured on magnetic resonance images. World J Clin Cases.

